# Brain-Derived Neurotrophic Factor as a Potential Mediator of the Beneficial Effects of Myo-Inositol Supplementation during Suckling in the Offspring of Gestational-Calorie-Restricted Rats

**DOI:** 10.3390/nu16070980

**Published:** 2024-03-27

**Authors:** Ana Valle, Pedro Castillo, Adrián García-Rodríguez, Andreu Palou, Mariona Palou, Catalina Picó

**Affiliations:** 1 Laboratory of Molecular Biology, Nutrition and Biotechnology (Group of Nutrigenomics, Biomarkers and Risk Evaluation), University of the Balearic Islands, 07122 Palma, Spain; 2 Health Research Institute of the Balearic Islands (IdISBa), 07010 Palma, Spain; 3 Artificial Intelligence Research Institute of the Balearic Islands (IAIB), 07122 Palma, Spain; 4 CIBER de Fisiopatología de la Obesidad y Nutrición (CIBEROBN), 28029 Madrid, Spain

**Keywords:** metabolic programming, insulin sensitivity, glucose control, hypothalamus, adipose tissue, browning, perinatal, BDNF

## Abstract

This study aims to investigate the potential mechanisms underlying the protective effects of myo-inositol (MI) supplementation during suckling against the detrimental effects of fetal energy restriction described in animal studies, particularly focusing on the potential connections with BDNF signaling. Oral physiological doses of MI or the vehicle were given daily to the offspring of control (CON) and 25%-calorie-restricted (CR) pregnant rats during suckling. The animals were weaned and then fed a standard diet until 5 months of age, when the diet was switched to a Western diet until 7 months of age. At 25 days and 7 months of age, the plasma BDNF levels and mRNA expression were analyzed in the hypothalamus and three adipose tissue depots. MI supplementation, especially in the context of gestational calorie restriction, promoted BDNF secretion and signaling at a juvenile age and in adulthood, which was more evident in the male offspring of the CR dams than in females. Moreover, the CR animals supplemented with MI exhibited a stimulated anorexigenic signaling pathway in the hypothalamus, along with improved peripheral glucose management and enhanced browning capacity. These findings suggest a novel connection between MI supplementation during suckling, BDNF signaling, and metabolic programming, providing insights into the mechanisms underlying the beneficial effects of MI during lactation.

## 1. Introduction

Gestation and lactation are crucial stages of development that exert a significant impact on future metabolic health [1,2,3,4]. Both epidemiological studies and pre-clinical research in animal models have shown that unfavorable conditions experienced during gestation, particularly during early pregnancy, can alter offspring programming, making them more prone to accumulating excess body weight and suffering metabolic alterations, such as insulin resistance, later in life [2,5,6]. In contrast, optimal lactational conditions, as represented in humans by maternal milk compared to infant formula feeding, are protective and can potentially reverse the alterations caused by adverse pregnancy conditions [7,8,9]. Although the precise mechanisms behind these benefits are not yet fully known, certain bioactive compounds present in breast milk, like leptin, have been proposed to play a biological role in shaping metabolic health and contributing to the protective effects of breastfeeding [10]. In rats, it has been demonstrated that leptin supplementation, at physiological doses, during the lactation period prevents the development of obesity and related disorders in later life [11]. Additionally, it has been found to be effective in reversing most of the offspring alterations induced by gestational calorie restriction [12]. Besides leptin, other milk compounds may also be of relevance. In this context, previous studies have pointed to myo-inositol (MI), a natural component of breast milk, as a potentially relevant factor [13,14]. Breast milk contains a higher concentration of MI compared to the levels found in commercial infant formulas [15,16]. It has been reported that MI supplementation at physiological doses during lactation in male rats improved their metabolic health and prevented the development of insulin resistance and hypertriglyceridemia, which were programmed by 25%-maternal-calorie restriction during pregnancy [13]. This effect was particularly evident when the rats were exposed to an obesogenic environment in adulthood [13]. One of the mechanisms contributing to the observed beneficial effects of MI involves its potential to reverse the early alterations in hypothalamic structure and function caused by gestational calorie restriction, exhibiting similarities to the effects observed with leptin [17]. However, it is yet to be determined which other targets of MI could account for the increased insulin sensitivity in adulthood of the animals supplemented with MI during suckling, particularly in the offspring of the dams that underwent calorie restriction.

Brain-derived neurotrophic factor (BDNF) is a multifaceted neurotrophin that is involved in the regulation of nerve growth and synaptic plasticity [18]. BDNF and its high-affinity receptor, neurotrophic receptor tyrosine kinase 2 (NTKR2), are abundant in the brain, as well as in peripheral tissues, such as the skeletal muscle, liver, pancreas, adipose tissue, and immune cells [19,20]. Thus, in addition to its role in cognition, it also serves as a pleiotropic signaling molecule, with implications for regulating energy balance [19,20,21]. Concretely, BDNF acts as a metabolism regulator and is essential in the management of the adaptive responses of the whole body to changes in energy intake and energy expenditure [20]. It connects the central nervous system to peripheral tissues, including the adipose tissue, muscle, and liver [20]. Among other effects, it regulates thermogenesis in brown adipose tissue (BAT), induces white adipose tissue (WAT) browning [22], and modulates insulin sensitivity and glucose uptake in skeletal muscle and adipose tissues [23]. Hence, an improvement in BDNF signaling could mediate, to a certain extent, the effects of MI in the offspring of dams exposed to gestational calorie restriction, associated with the better management of energy metabolism. Therefore, in the present study, we aimed to evaluate the short- and long-term effects of gestational calorie restriction and the potential benefits of MI supplementation during lactation on central and peripheral BDNF signaling, focusing on improved later metabolic health, particularly ameliorated insulin sensitivity, in male and female rats.

## 2. Materials and Methods

### 2.1. Animals

The Bioethics Committee of the University of the Balearic Islands authorized the animal protocol (Resolution Number 2018/13/AEXP) used in the present study, guaranteeing the ethical standards and animal care guidelines.

Wistar rats (Charles River Laboratories, Barcelona, Spain) were housed at 22 °C with a 12-h-light/12-h-dark cycle and given free access to tap water and fed a standard chow diet (Panlab, Barcelona, Spain; 3.3 kcal/g, 8.0% of calories from fat), unless stated otherwise.

**Figure 1:**
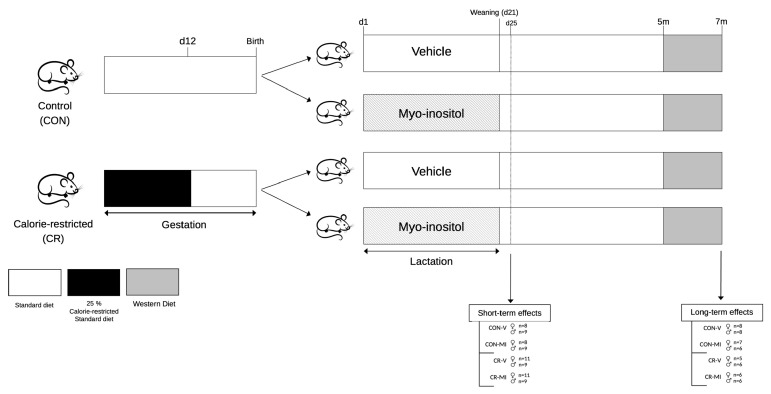
Schematic representation of the experimental design. Abbreviations: controls (CON), calorie-restricted (CR), vehicle (V), myo-inositol (MI), day (d), months (m).

Figure 1 represents the animal design followed for the present work. The study involved male and female rats, divided into the following four groups: CON-V, CON-MI, CR-V, and CR-MI, as previously described [13]. Female virgin rats were paired with male control rats. When pregnant, the females were classified into CON (*n* = 9) and CR (*n* = 11) dams. From 1 to 12 days of gestation, the CR dams were subjected to 25% calorie restriction, followed by ad libitum feeding for the rest of the gestation period. This period of energy restriction was selected according to epidemiological evidence and animal studies indicating that the early stages of gestation are particularly pivotal in programming later predisposition to obesity in offspring [2,24].

After delivery (day 1), the litters were adjusted to 10 pups per dam, with 5 females and 5 males when possible. During lactation, the pups in both the CON and CR litters received either MI (CON-MI and CR-MI) or the vehicle (CON-V and CR-V) daily through a pipette, while the dams were fed ad libitum. MI (Sigma-Aldrich, Saint Louis, MO, USA) was mixed with water. The daily oral dose administered was twice that of the average quantity derived from maternal milk. This calculation was based on the mean concentration of MI in the milk of the control dams at different stages of lactation (0.426 mg/mL) [14] and the estimated daily amount of milk consumed by the pups (from 0.5 mL on postnatal day 1 to 11.88 mL on postnatal day 20) [25], and the daily doses of MI supplemented throughout the lactation period were calculated. Thus, the daily doses of MI supplemented during the 20-day lactation period were as follows: 0.43, 0.85, 1.28, 1.70, 2.13, 2.67, 3.20, 3.73, 4.26, 4.80, 5.33, 5.86, 6.39, 6.93, 7.46, 7.99, 8.52, 9.06, 9.59, and 10.12 mg. The pups were weaned on day 21 of lactation, housed in pairs, and fed an SD.

Study 1. Short-term effects of myo-inositol supplementation during suckling in the offspring of gestational-calorie-restricted rats

On postnatal day 25, one set of animals from each group (*n* = 8–11 per group) was decapitated within the first 2 h of the light cycle under fed conditions. Blood samples were collected from the neck, and hypothalamus, retroperitoneal and inguinal white adipose tissue depots (rWAT and iWAT, respectively), and brown adipose tissue depot (BAT) were also collected, weighed, and stored at −80 °C for further analysis.

Study 2. Long-term effects of myo-inositol supplementation during lactation in the offspring of gestational-calorie-restricted rats

Another set of animals (*n* = 5–8 rats per group) was monitored until reaching 7 months of age. These rats were fed an SD until 5 months of age. Blood was collected from the saphenous vein under fed conditions and without anesthesia. The animals were then switched to a high-fat, high-sugar diet, known as a Western diet (WD; Research Diets, New Brunswick, NJ, USA; 4.7 kcal/g, 40.0% fat, 43.0% carbohydrate, 17.0% protein), for 2 months. At 7 months of age, the rats were sacrificed under fed conditions by decapitation, and blood samples were collected from the neck. Subsequently, the rWAT, iWAT, BAT, and hypothalamus were excised, weighed, and stored at −80 °C for further analysis.

### 2.2. BDNF Quantification by Enzyme-Linked Immunosorbent Assay (ELISA)

The BDNF levels were measured in plasma samples from the animals at three time points (25 days, 5 months, and 7 months of age). The blood collected was centrifuged at 1000× *g* and 4 °C for 10 min to obtain the plasma samples. An ELISA kit (#ERBDNF, Invitrogen, Carlsbad, CA, USA) was used to determine the circulating BDNF levels.

### 2.3. Gene Expression Analyses

RNA was isolated from the hypothalamus and iWAT with the Tripure Reagent (Roche Diagnostic Gmbh, Mannheim, Germany) and from the rWAT and BAT with the E.Z.N.A. RNA Kit I (Omega Bio-Tek, Inc., Norcross, GA, USA), in accordance with the respective instructions of the manufacturers. The total RNA extracted from all tissues was quantified using a NanoDrop ND-100 spectrophotometer (NanoDrop Technologies, Inc., Wilmington, DE, USA), and 1% agarose gel electrophoresis was used to assess the integrity.

Next, the mRNA expression levels of specific genes in different adipose depots (BAT, rWAT and iWAT) and the hypothalamus were quantified using real-time polymerase chain reaction in both 25-day-old and 7-month-old animals, following the same procedure as previously described [13].

The genes whose mRNA expression levels were analyzed in each tissue and age were as follows: at 25 days and 7 months, brain-derived neurotrophic factor (*Bdnf*) and neurotrophic receptor tyrosine kinase 2 (*Ntkr2*) in the hypothalamus, rWAT, iWAT, and BAT; at 25 days, insulin receptor (*Insr*) and leptin receptor (*Lepr*) in the four tissues studied and uncoupling protein 1 (*Ucp1*) in the BAT; at 25 days and 7 months, agouti-related protein (*Agrp*), cocaine- and amphetamine-regulated transcript (*Cart*), melanocortin 4 receptor (*Mc4r*), neuropeptide Y (*Npy*), and pro-opiomelanocortin (*Pomc)* in the hypothalamus; at 7 months, cell-death-inducing DFFA-like effector A (*Cidea*), *Ucp1*, and solute carrier family 2 member 4 (*Slc2a4*) in the three adipose depots and *Insr* and *Lepr* in the hypothalamus; and at 7 months, hexokinase (*Hk*) and homeobox C9 (*Hox-c9*) in the rWAT, iWAT, and BAT. Housekeeping genes, *ß-actin*, guanosine diphosphate dissociation inhibitor 1 (*Gdi1*), and/or low-density lipoprotein receptor-related protein 10 (*Lrp10*) were used. 

### 2.4. Protein Expression Analyses

The UCP1 levels in the BAT and GLUT4 in the iWAT of 7-month-old rats were determined using Western blot analysis. Tissue samples were disrupted by homogenization in RIPA buffer containing a cocktail of protease and phosphatase inhibitors (Thermo Fisher, Rockford, IL, USA) at 4 °C using a weight-to-volume ratio of 1:5. To obtain the supernatant, which was used for protein analysis, the homogenate was centrifuged at 7500× *g* for 2 min at 4 °C. The quantification of total protein was performed with a BCA protein assay kit (Pierce, Rockford, IL, USA). 

Then, 10 µg and 75 µg of total protein for BAT and iWAT, respectively, were processed and separated utilizing 10% SDS-polyacrylamide gel electrophoresis, as previously described [13]. A rabbit polyclonal anti-UCP1 antibody (GTX10983, GeneTex, Irvine, CA, USA) and a mouse polyclonal anti-GLUT4 antibody (#2213, Cell Signaling Technology, Danvers, MA, USA) were used. An Odyssey Infrared Imaging System (Li-COR Biosciences, Lincoln, NE, USA) was employed for analysis. β-actin and/or HSP90 (#3700 and #4877, respectively, Cell Signaling Technology) were the reference proteins.

### 2.5. Statistical Analysis

Data are presented as mean ± SEM. To ascertain the impact of gestational calorie restriction (R), MI treatment during lactation (M), and/or sex (S), two- or three-way analyses of variances (ANOVA) were utilized. Individual group comparisons were conducted via Student’s *t*-test. Before conducting the analyses, Shapiro–Wilk and Levene tests were carried out to check normality and homogeneity, respectively. If required, data were logarithmically transformed. Statistical significance was defined as a threshold of *p* < 0.05. Statistics were conducted with SPSS for Windows version 22 (SPSS, Chicago, IL, USA). 

## 3. Results

### 3.1. Weight-Related Parameters

Table 1 and Table 2 present weight-related parameters for the offspring of the control and CR dams, supplemented either with MI or the vehicle, at the ages of 25 days and 7 months, respectively. At the age of 25 days, the offspring of the dams exposed to gestational calorie restriction showed a lower body weight (two-way ANOVA), as well as lower body fat content and weights of the rWAT and iWAT (three-way ANOVA). Non-significant effects of MI supplementation were found.

At 7 months of age, no significant differences were found in the body weight and body fat content due to maternal conditions or MI treatment, as previously described [13]. No differences were found regarding the weights of the rWAT and iWAT depots either. However, concerning the BAT, the CR males, irrespective of treatment with MI, exhibited a lower weight compared to their controls, whereas the CR females showed a higher weight than their respective controls (two-way ANOVA). Additionally, among the females, CR-MI displayed a greater BAT weight than CR-V (Student’s *t*-test).

### 3.2. Circulating BDNF

**Figure 2:**
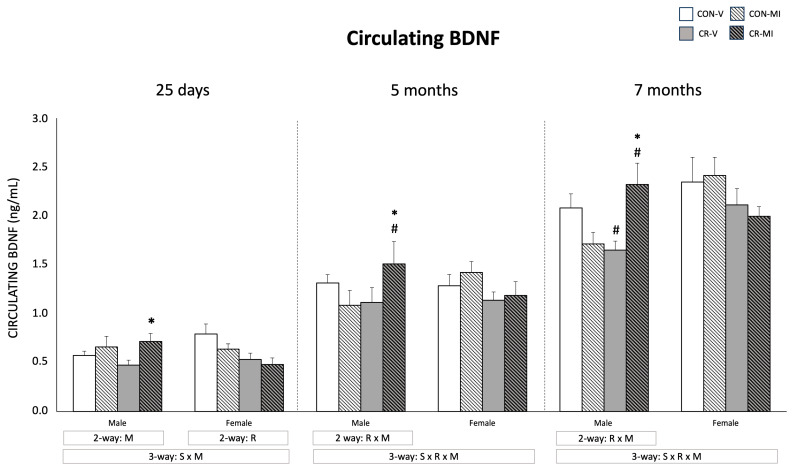
Plasma BDNF levels of male and female rats at 25 days, 5 months, and 7 months of age. Data are means ± SEM. Statistics: Three-way ANOVA was conducted to investigate the impacts of gestational calorie restriction (R), myo-inositol treatment (M), and/or sex (S). Within each sex, two-way ANOVA was used to assess the effects of R and/or M. Student’s *t*-test was used for individual group comparisons: *, different from their corresponding vehicle-supplemented group; #, different compared to their corresponding control group. Abbreviations: control (CON), gestational calorie-restricted (CR), vehicle (V), myo-inositol (MI).

The circulating levels of BDNF are shown in Figure 2. At 25 days, the male animals supplemented with MI during lactation exhibited greater BDNF levels compared to the vehicle group (two-way ANOVA), with the differences being more marked and significant by the Student’s t-test in the CR animals. This trend was maintained in adulthood in the case of the CR males that were supplemented with MI, and an interactive effect between calorie restriction and MI supplementation was observed in the adult male animals (two-way ANOVA). Concretely, at the age of 7 months, the CR-V males displayed lower circulating BDNF levels than the CON-V, whereas, at 5 and 7 months, the MI-treated CR males, but not the CON males, showed higher BDNF levels than the vehicle-treated ones (Student’s *t*-test). Unlike the males, no significant effects of MI supplementation were observed in the females, and an interactive effect was observed between sex and MI at 25 days, and between sex, maternal calorie restriction, and MI at the ages of 5 and 7 months, when considering males and females (three-way ANOVA). Of note, at the age of 25 days, the CR females displayed lower circulating BDNF levels than the controls (two-way ANOVA), but significant differences were not maintained in adulthood.

### 3.3. Gene and Protein Expression Levels

**Figure 3:**
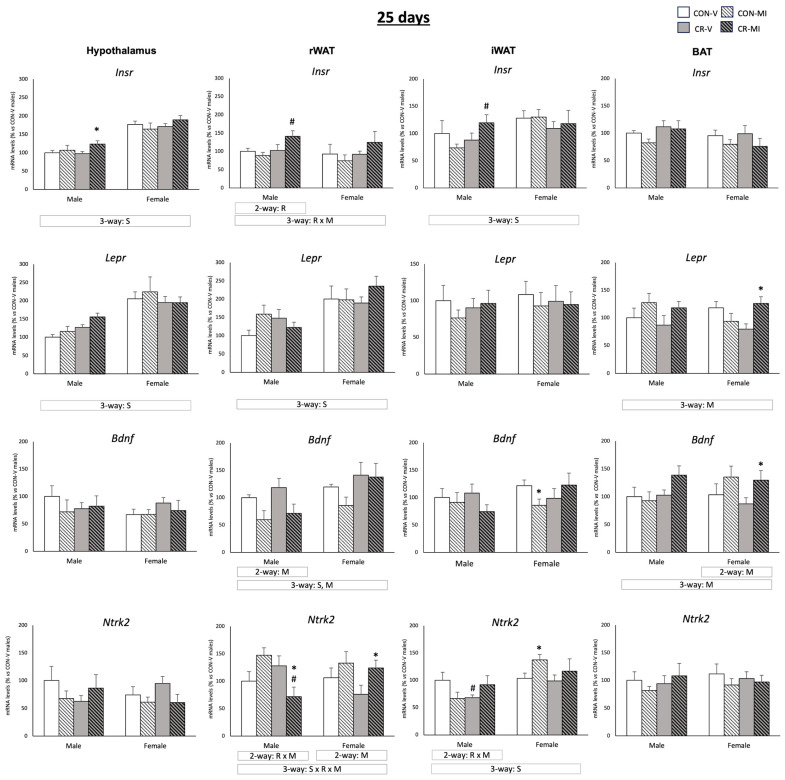
Expression levels of *Insr*, *Lepr*, *Bdnf,* and *Ntrk2* genes in the hypothalamus, retroperitoneal and inguinal white adipose tissue (rWAT and iWAT), and brown adipose tissue (BAT) of 25-day-old offspring from controls (CON) and calorie-restricted (CR) dams during gestation, treated with either vehicle (V) or myo-inositol (MI) during the lactation period. mRNA levels are expressed as a percentage of the value for the CON-V male rats. Data are mean ± SEM. Statistics: Three-way ANOVA was conducted to investigate the impacts of gestational calorie restriction (R), lactational myo-inositol treatment (M), and/or sex (S). Within each sex, two-way ANOVA was used to assess the effects of R and/or M. Student’s *t*-test was used for individual group comparisons: *, different from their corresponding vehicle-supplemented group; #, different compared to their corresponding control group.

Figure 3 shows the mRNA expression levels of *Insr*, *Lepr*, *Bdnf,* and *Ntrk2* at 25 days. MI supplementation resulted in greater *Insr* mRNA levels in the hypothalamus of the CR male animals in comparison to CR-V (Student’s *t*-test). Furthermore, the CR-MI animals exhibited higher *Insr* expression in both the retroperitoneal and inguinal WAT depots compared to the CON-MI animals (Student’s *t*-test). The *Bdnf* mRNA levels in the rWAT were reduced by the MI treatment (three-way ANOVA), especially in the males (two-way ANOVA). Additionally, the CR-MI females had elevated *Bdnf* mRNA levels in the rWAT in comparison to the CON-MI females (Student’s *t*-test). In the iWAT, the CON-MI females displayed lower mRNA levels of *Bdnf* than the vehicle-treated controls (Student’s *t*-test). The MI treatment also led to an upregulation of *Ntrk2* mRNA expression in the rWAT of the male controls (*p* = 0.053, Student’s *t*-test), whereas it led to a decreased expression in the CR males compared to their vehicle-treated counterparts (two-way ANOVA, interactive effect between calorie restriction and MI supplementation; and Student’s *t*-test). In the iWAT, the CR males treated with the vehicle, but not those treated with MI, displayed lower mRNA levels of *Ntrk2* than their controls (Student’s *t*-test). In the females, *Ntrk2* mRNA expression in the rWAT and iWAT (the latter only in the CON animals) increased due to MI supplementation compared to the vehicles (two-way ANOVA, Student’s *t*-test). In the BAT, MI supplementation also resulted in higher mRNA levels of *Bdnf* and *Lepr* in comparison to the vehicle-treated counterparts (three-way ANOVA). Of note, the expression levels of the aforementioned genes were different depending on the sex of the animals. The female rats displayed higher expression levels of *Insr* (in the hypothalamus and iWAT), *Lepr* (in the hypothalamus and rWAT), *Bdnf* (in the iWAT), and *Ntrk2* (in the iWAT) than the males (three-way ANOVA). 

**Figure 4:**
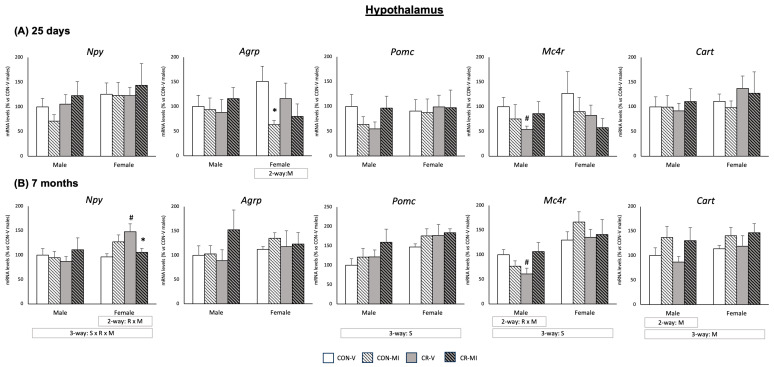
Expression levels of hypothalamic energy control genes of 25-day-old and 7-month-old offspring from controls (CON) and calorie-restricted (CR) dams during gestation, treated with either vehicle (V) or myo-inositol (MI) during the lactation period. mRNA levels are expressed as a percentage of the value for the CON-V male rats. Data are mean ± SEM. Statistics: Three-way ANOVA was conducted to investigate the impacts of gestational calorie restriction (R), lactational myo-inositol treatment (M), and/or sex (S). Within each sex, two-way ANOVA was used to assess the effects R and/or M. Student’s *t*-test was used for individual group comparisons: *, different from their corresponding vehicle-supplemented group; #, different compared to their corresponding control group.

Figure 4 depicts the gene expression profiles of key hypothalamic energy control genes in the studied animal groups at both 25 days and 7 months of age. These genes were selected according to their key function in the control of feeding behavior and energy homeostasis. Moreover, previous studies have documented changes in their mRNA expression due to maternal energy restriction and/or to leptin/MI supplementation during the suckling period [17,26]. At 25 days (Figure 4A), the MI supplementation during suckling resulted in decreased mRNA levels of the orexigenic peptide *Agrp* in the females, which was more marked and only significant by Student’s *t*-test in the CR animals. mRNA expression of the anorexigenic peptide *Pomc* exhibited a different expression pattern depending on the maternal conditions and lactational treatment in the males, with a trend to decrease in the CR-V animals compared to the CON-V, but to increase in the CR-MI compared to the CON-V (interactive effect between calorie restriction and MI supplementation, *p* = 0.059, two-way ANOVA). Notably, MI supplementation also counteracted the significant downregulation of *Mc4r* mRNA expression displayed by the CR-V males compared to the CON-V (Student’s *t*-test). Neither the maternal conditions nor MI supplementation resulted in significant changes in *Npy* or *Cart* mRNA levels in the hypothalamus of the male and female animals at 25 days. Of note, at 7 months, the female rats showed greater expression levels of *Pomc* and *Mc4R* than the males (three-way ANOVA).

At 7 months of age (Figure 4B), both male and female rats displayed increased Cart mRNA levels due to MI supplementation (two-way ANOVA), regardless of the maternal conditions. Notably, in the males exposed to CR, MI supplementation counteracted the significant downregulation of *Mc4r* mRNA expression found in the CR-V males (Student’s *t*-test), similar to that observed at 25 days. In the females, MI supplementation mitigated the elevation in the mRNA expression of the orexigenic peptide Npy induced by maternal calorie restriction (Student’s *t*-test). No significant effects of maternal conditions or MI supplementation were observed on the mRNA levels of *Agrp* and *Pomc* in the animals at 7 months. The mRNA levels of *Insr* and *Lepr* in the hypothalamus of the 7-month-old rats were also analyzed, but no significant differences were observed due to maternal conditions or the supplementation with MI during lactation (Supplementary Figure S1).

**Figure 5:**
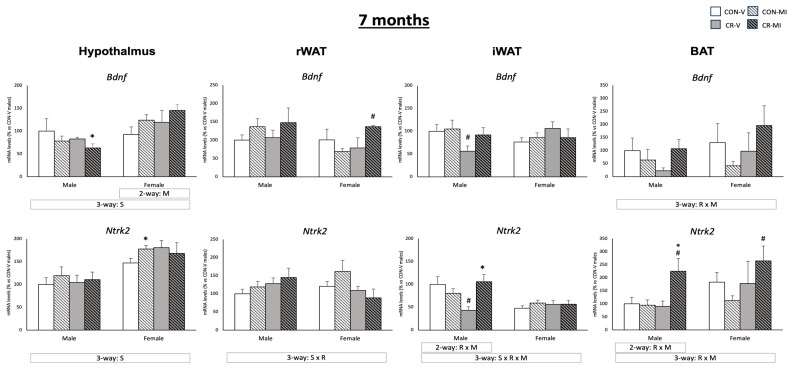
Expression levels of *Bdnf* and *Ntrk2* genes in the hypothalamus, retroperitoneal and inguinal white adipose tissue (rWAT and iWAT), and brown adipose tissue (BAT) of 7-month-old offspring from controls (CON) and calorie-restricted (CR) dams during gestation, treated with either vehicle (V) or myo-inositol (MI) during the lactation period. mRNA levels are expressed as a percentage of the value for the CON-V male rats. Data are mean ± SEM. Statistics: Three-way ANOVA was conducted to investigate the impacts of gestational calorie restriction (R), lactational myo-inositol treatment (M), and/or sex (S). Within each sex, two-way ANOVA was used to assess the effects of R and/or M. Student’s *t*-test was used for individual group comparisons: *, different from their corresponding vehicle-supplemented group; #, different compared to their corresponding control group.

Figure 5 shows *Bdnf* and *Ntrk2* mRNA levels in the four tissues studied at the age of 7 months. In the hypothalamus, MI supplementation led to an upregulation of *Bdnf* mRNA expression in the females (two-way ANOVA), while, in the CR males, it resulted in reduced *Bdnf* mRNA levels (Student’s *t*-test). Of note, the *Bdnf* and *Ntrk2* expression levels in the hypothalamus were higher in the females than in the males (three-way ANOVA). In the BAT, *Bdnf* exhibited a different expression pattern depending on the maternal conditions and lactational treatment (interactive effect, three-way ANOVA), with a trend to decrease in the CR animals treated with the vehicle in comparison to their controls, but to increase in the CR animals when treated with MI. An interactive effect between both conditions was also found for the mRNA levels of *Ntrk2* in the iWAT (only males) and in the BAT (males and females) (two- and three-way ANOVA, respectively). Specifically, in the iWAT, the CR males supplemented with MI exhibited greater mRNA expression of *Ntrk2* compared to their vehicle-treated counterparts, and, in the case of the BAT, also compared to the MI-treated controls (Student’s *t*-test). Of note, in the iWAT, the CR males treated with the vehicle experienced a reduction in *Bdnf* and *Ntrk2* mRNA levels compared to their controls (Student’s *t*-test). In the rWAT, the CR females supplemented with MI displayed an increased mRNA expression of *Bdnf* than their controls (Student’s *t*-test).

**Figure 6:**
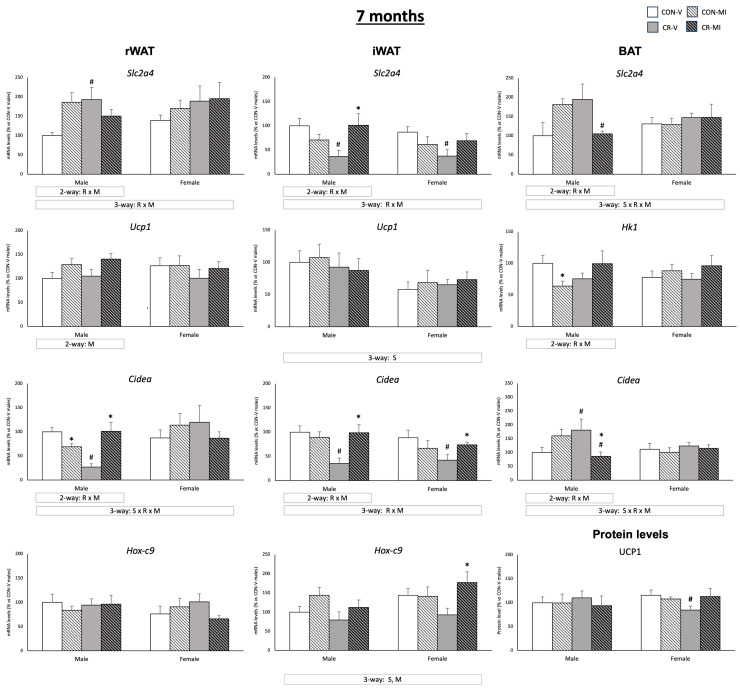
Expression levels of genes implicated in glucose uptake, browning, and thermogenesis in three adipose tissues (retroperitoneal and inguinal white adipose tissue (rWAT and iWAT, respectively) and brown adipose tissue (BAT)), as well as UCP1 protein levels in BAT, of 7-month-old offspring from controls (CON) and calorie-restricted (CR) dams during gestation, treated with either vehicle (V) or myo-inositol (MI) during the lactation period. Protein levels are expressed as a percentage of the value for the CON-V male rats. mRNA levels are expressed as a percentage of the value of the CON-V male animals. Data are mean ± SEM. Statistics: Three-way ANOVA was conducted to investigate the impacts of gestational calorie restriction (R), lactational myo-inositol treatment (M), and/or sex (S). Within each sex, two-way ANOVA was used to assess the effects of R and/or M. Student’s *t*-test was used for individual group comparisons: *, different from their corresponding vehicle-supplemented group; #, different compared to their corresponding control group. Representative bands from Western blot analysis of UCP1 and reference proteins (β-Actin and HSP90) for each animal group are displayed in Figure S3.

Figure 6 shows the mRNA expression levels of genes related to glucose uptake, browning, and/or thermogenesis in the three adipose depots studied, along with the UCP1 protein levels in the BAT for the animal groups studied at the age of 7 months. Different expression patterns of *Slc2a4* were observed in the three adipose depots analyzed, depending on the gestational conditions and the MI treatment during lactation (three-way ANOVA). While the *Slc2a4* mRNA levels tended to increase in the controls supplemented with MI in the rWAT and BAT, they did not change (in rWAT), or were reduced (in BAT) in, the CR rats supplemented with MI. In the iWAT, the CR animals (both males and females) treated with the vehicle exhibited lower *Slc2a4* mRNA levels compared to their controls (Student’s *t*-test). Interestingly, this decrease was not observed in the CR animals that were treated with MI, and the CR-MI males showed higher mRNA expression levels than the CR-V (Student’s *t*-test). On the other hand, the *Hk1* mRNA levels were decreased in the CON-MI with respect to the CON-V in the BAT (Student’s *t*-test). With respect to the browning- and thermogenesis-related genes, MI supplementation resulted in higher *Ucp1* (males) and *Hox-c9* (males and females) mRNA levels in the rWAT and iWAT, respectively (two- and three-way ANOVA, respectively) and counteracted the decrease in the UCP1 protein levels observed in the BAT of the CR females (Student’s *t*-test). The gene expression pattern of *Cidea* in the three depots was dependent on the maternal conditions and MI supplementation (interactive effect by three- or two-way ANOVA). Notably, a reduction in the *Cidea* mRNA levels was observed due to gestational calorie restriction in the rWAT (only males) and the iWAT (males and females), compared to their controls, and this reduction was reversed with MI supplementation (Student’s *t*-test). In contrast, the *Cidea* expression levels in the BAT were increased in the CR-V males compared to their controls (Student’s *t*-test), while the CR-MI males exhibited reduced expression compared to the CR-V and CON-MI males (Student’s *t*-test). In the iWAT, sex differences were observed in the expression levels of *Ucp1* and *Hox-c9*, with the females exhibiting lower expression levels of *Ucp1* and higher levels of *Hox-c9* in comparison with the males (three-way ANOVA). The mRNA levels of *Ucp1* in the BAT of the 25-day-old and 7-month-old rats are depicted in Supplementary Figure S2. At the age of 25 days, the CR females treated with the vehicle exhibited lower mRNA levels of *Ucp1* than their controls (Student’s *t*-test). However, no significant differences were found at 7 months of age that were attributable to the gestational conditions or MI supplementation during lactation.

**Figure 7:**
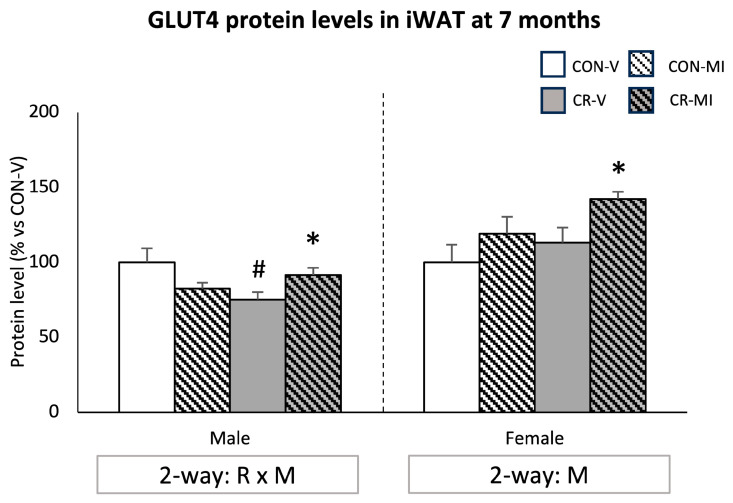
Protein levels of glucose transporter 4 (GLUT4) in the inguinal white adipose tissue of 7-month-old offspring from controls (CON) and calorie-restricted (CR) dams during gestation, treated with either vehicle (V) or myo-inositol (MI) during the lactation period. Data are mean ± SEM, expressed as the percentage for the CON-V animals for each sex. Statistics: Two-way ANOVA was used to assess the effects of calorie restriction (R) and/or myo-inositol supplementation (M). Student’s *t*-test was used for individual group comparisons: *, different from their corresponding vehicle-supplemented group; #, different compared to their corresponding control group. Representative bands from Western blot analysis of GLUT4 and reference proteins (β-Actin and/or HSP90) for each animal group are displayed in Supplementary Figure S3.

Figure 7 shows the GLUT4 protein levels in the iWAT at the age of 7 months. The CR male animals treated with the vehicle showed a decrease in GLUT4 protein levels compared to their controls, whereas MI supplementation in this group led to increased levels (Student’s *t*-test). In the females, MI supplementation resulted in greater GLUT4 levels compared to their respective vehicle-treated controls (two-way ANOVA). This difference was more pronounced and significant by Student’s *t*-test in the CR females.

## 4. Discussion

Maternal calorie restriction during pregnancy, even if mild or moderate (20–25%), has been described to result in a higher predisposition of the offspring to suffer from metabolic disorders in adulthood, such as obesity and type 2 diabetes, especially if exposed to the stress of an unbalanced diet [2]. The mechanisms and factors underlying the malprogramming effects due to these maternal conditions seem to be multifactorial but are not completely elucidated. They involve hypothalamic alterations, impaired sympathetic drive, and dysregulations of glucose homeostasis, among others [2]. Most of them can be reverted, at least in part, by leptin intake during lactation [10]. MI is also a natural component of maternal milk [15,16,27]. Evidence from animal studies suggests that increased MI intake during lactation may protect against the detrimental effects of gestational energy restriction [13,17]. Specifically, supplementing with physiological doses of MI in neonate rats during lactation protects them against the alterations in the structure and function of the hypothalamus and the development of insulin resistance caused by 25% maternal calorie restriction during the 12 days of gestation, in a sex-dependent manner [13,17]. Here, we present novel findings that unveil a potential link between MI supplementation during lactation and the modulation of BDNF signaling, which is particularly noted under conditions of mild gestational calorie restriction. This is supported by evidence of the promotion of anorexigenic pathways in the hypothalamus, the upregulation of browning markers, and enhanced glucose uptake capacity in adipose tissues. Therefore, BDNF emerges as a plausible contributor to the effects of MI ingested during lactation and its potential benefits on later metabolic health, with a specific focus on improving glycemic control. 

The effects of chronic MI treatment on glucose homeostasis have been previously reported [28,29,30,31,32,33,34,35]. Studies on both humans and rodents show that treatment with MI during pregnancy significantly improves the alterations associated with gestational diabetes, leading to a reduction in the number of pregnant women requiring insulin treatment and ameliorating their fetus’s development [28,29,30,31,32]. Other animal studies have also shown that MI treatment may improve insulin sensitivity [33,34,35]. Notably, in the present study, a sex-dependent effect was observed. The male offspring (but not the female) of the calorie-restricted dams that were treated with MI during lactation displayed, at a juvenile age, increased expression levels of the *Insr* gene in the hypothalamus and in two WAT depots, with respect to the other groups. This effect was observed before the detrimental outcomes of the adverse maternal conditions became apparent. This could be interpreted as an improved capacity of MI-treated animals, particularly males, to respond to insulin in early life, in accordance with the insulin-sensitivity-enhancing effect of MI [33,34,35]. In fact, we have previously reported that these male CR animals that were supplemented with MI during the suckling period displayed, in adulthood and under obesogenic nutritional conditions, improved circulating insulin levels under fed conditions, HOMA-IR index, and plasma lipid profile [13]. However, the mechanisms that are responsible for promoting this improvement were not fully established [17]. In this context, the substantial rise in circulating BDNF levels from the juvenile stage of life in those animals treated with MI, especially in the male offspring of the dams exposed to gestational calorie restriction, is noteworthy. BDNF plays a pivotal role in glycemic regulation, as evidenced by functional studies in animals showing a significant decrease in circulating glucose levels upon BDNF administration [36]. However, it should be noted that normoglycemic rats do not experience a BDNF-induced effect on blood glucose [36]. This observation partially explains the stimulation of early insulin responsiveness capacity following MI supplementation, which may account for enhanced insulin sensitivity, specifically in the CR males. Therefore, the increase in circulating BDNF levels triggered by MI supplementation during lactation may serve as one of the contributing factors to the observed improvement of early insulin responsiveness in these animals. This effect is particularly evident in the offspring exposed to adverse fetal conditions, where metabolic programming has been altered. 

The hypothalamus is crucial in regulating energy balance and feeding behavior by modulating the synthesis of neuropeptides and activating the sympathetic nervous system in response to diverse stimuli, including circulating hormones such as insulin and leptin [26]. In the juvenile CR-MI males, the upregulation of *Insr* mRNA expression in the hypothalamus was accompanied by higher circulating levels of BDNF, which was concurrent with a normalization of *Mc4r* expression to values of the control group, since the vehicle-treated CR males showed decreased levels. MC4R plays a central role in controlling energy balance, influencing glucose and lipid metabolism, and coordinating sympathetic activity [37,38]. Rodent studies suggest a significant potentiating effect of MC3/4R on insulin-mediated glucose disposal, regardless of changes in food intake and body weight [39,40]. Furthermore, a decrease in circulating insulin levels has been found after the stimulation of the melanocortin pathway in the hypothalamus of mice [41]. Of note, the expression of the *Pomc* gene, which encodes for a precursor of the α-melanocyte-stimulating hormone (α-MSH), the ligand of MC4R [42], showed a similar trend to that of *Mc4r*. Altogether, the gene expression patterns of anorexigenic hypothalamic effectors may contribute to the enhanced insulin sensitivity at the central level in the offspring of gestational-calorie-restricted dams that were treated with MI, in comparison with those treated with the vehicle. Therefore, the current results suggest a relationship between circulating BDNF and hypothalamic gene expression, although direct evidence is not provided. It is worth mentioning that BDNF has been suggested to exhibit an anorexigenic action, and a lack of BDNF has been related to increased food intake and reduced energy expenditure [43]. Furthermore, a hypothalamic leptin–BDNF axis has been proposed, with leptin-inducing BDNF action [43,44,45]. In this line, previous research has suggested a potential neurotrophic action of MI when supplemented at physiological doses during the suckling period in the offspring of energy-restricted pregnant dams [17]. This effect is comparable, to some extent, to that observed with leptin supplementation [17]. This was tentatively attributed, in part, to a potential role of MI in enhancing the activity of leptin naturally ingested with milk [17]. Considering the documented role of BDNF in the developing brain, wherein it stimulates neuron growth and survival [44], and the link between leptin and BDNF [43,44,45], it is plausible to propose BDNF as a mechanism contributing to the neurotrophic action of MI ingested during lactation—at least in males. 

Unlike the males, the females that were supplemented with MI during suckling did not show any significant differences in their circulating levels of BDNF at any of the life stages studied when compared to those receiving the vehicle. However, the female animals, both the controls and the CR, showed, at a juvenile age, a reduced hypothalamic expression of the orexigenic effector AGRP, a known MC3/4R antagonist [13,17,46]. These results agree with a plausible role of MI ingested during the suckling period in promoting anorexigenic pathways at the central level through a leptin-sensitizing action. Hence, MI may exert its effects in a sex-specific manner, as previously suggested [13,17], and potentially involve different mechanisms. Of note, the regulation of central *Bdnf* expression and circulating BDNF levels has been proposed to be influenced by diet and energy status in a sex-dependent manner [47,48]. This observation also supports the proposed sex differential mechanisms mediating the effects of MI ingested during the suckling period. 

In adulthood, the elevated circulating levels of BDNF persisted in the CR males that received MI treatment during suckling. This was coincident with a reduction in the *Bdnf* expression levels in the hypothalamus of these animals. These results could be tentatively attributed to the presence of a negative feedback loop mechanism in response to the increased circulating levels of BDNF, involving the activation of the expression of miRNAs in the brain, which, in turn, downregulate *Bdnf* expression [49]. Notably, this signaling pathway has been described to operate within a balanced state, ensuring that the BDNF protein levels stay within an ideal functional range. However, it is disrupted under certain central nervous system pathologies, such as Alzheimer’s disease [49]. In support of this postulation, the CON and CR female rats treated with MI, whose BDNF circulating levels did not differ from those of the rats treated with the vehicle, showed increased expression levels of *Bdnf* in the hypothalamus. This would also be in line with the propounded link between MI and BDNF. Moreover, the MI treatment also led to an increase in the expression of the anorexigenic peptide CART (controls and CR) and a reversal of the reduced *Mc4r* expression in the CR male animals. In the females, the MI treatment decreased the *Npy* expression levels in the CR animals, whose levels were increased by the effects of gestational calorie restriction. Taken together, these findings in the adult rats suggest that MI ingested at supplementary amounts during suckling leads to a potentiation of anorexigenic hypothalamic pathways over an orexigenic one. This effect is sex-dependent and largely counteracts the adverse effects associated with gestational calorie restriction. 

Adipose tissues, including both WAT and BAT, serve as critical organs in regulating energy balance and substrate metabolism [50]. They are also subject to peripheral hormone actions [19]. However, fat depots show strong morphological, secretory, and functional differences [51]. Consistently, maternal conditions or MI treatment during suckling were found to modulate the BDNF signaling pathway at the gene expression level in adipose depots, with differences depending on the depot, the age, and the sex of the animal. In the visceral depot studied here (rWAT), the diminished expression of *Bdnf* and its receptor of the male animals supplemented with MI found at a juvenile age may be the result of a feedback loop, as previously suggested at the central level, or due to the reduced adiposity, because of maternal energy restriction. In contrast, subcutaneous adipose depots, both iWAT and BAT, did not show such a decrease. Moreover, the MI treatment prevented the downregulation of BDNF receptor gene expression (*Ntrk2*) in the iWAT of the CR males and even led to a general stimulation of *Bdnf* expression in the BAT, maybe as a response to their incremented circulating levels of BDNF. Notably, the females supplemented with MI did not experience an increase in plasma BDNF levels compared to those treated with the vehicle. However, they did exhibit a compensatory upregulation of the expression of its receptor in both the rWAT and the iWAT (the latter only in the control group), along with an increase in *Bdnf* mRNA levels in the BAT at a juvenile age. In adulthood, the stimulated peripheral *Bdnf* signaling in the CR animals supplemented with MI was generally maintained in the adipose tissues, especially in the subcutaneous depots. Concretely, the MI treatment counteracted the decreased expression of *Bdnf* and *Ntrk2* occurring in the iWAT of the males of the CR group and resulted in increased *Ntrk2* expression in the BAT in the CR animals.

The peripheral role of BDNF is less characterized compared to its central action. BDNF has been proposed to increase the utilization of glucose in the muscle, liver, and BAT in obese and diabetic animals, while also increasing the levels of norepinephrine in these tissues [23]. This suggests a hypoglycemic effect of BDNF by a mechanism that involves central and peripheral pathways [23]. The effect of BDNF on the WAT is not well-defined but has been proposed to be related to the activation of browning [21] and energy expenditure [52]. Thus, in order to explore the potential targets affected by MI supplementation in relation to *Bdnf* signaling activation in adipose tissue, the expression of the key genes implicated in glucose uptake, and in browning and thermogenesis processes, were studied in three different adipose depots in adulthood. Interestingly, the iWAT showed the strongest influence of the MI treatment during suckling in the male offspring of the gestational-calorie-restricted dams. Subcutaneous WAT, the preeminent reservoir of triglycerides in the body, is characterized by reduced lipolytic activity but elevated responsiveness to insulin compared to visceral fat stores [53]. In this regard, the inguinal fat depot exhibited enhanced glucose uptake capacity, along with significant indicators of browning, in the MI-treated animals, particularly in the males and females exposed to adverse gestational conditions. Moreover, the MI treatment counteracted the decreased expression of genes related to these functions (*Slc2a4* and *Cidea*) occurring in the CR animals and of GLUT4 levels in the CR males. The recruitment of beige adipocytes has been shown to enhance metabolic status by promoting elevated glucose uptake from the bloodstream [54,55]. This suggests that the stimulation of browning in the iWAT can be partially responsible for the better glucose homeostasis of the CR males treated with MI during the suckling period. Furthermore, the mRNA expression of *Hox-c9* in the adipose tissue has been inversely related to obesity-related traits and positively associated with insulin sensitivity [56]. This is in line with the reported increase in adult insulin sensitivity after MI treatment in rats during the suckling period, particularly in the offspring of calorie-restricted dams [13]. Moreover, the rWAT has been identified as a markedly active visceral depot with a higher lipolytic and storage capacity compared to inguinal adipose tissue [57,58], which also displayed markers of beige adipocytes—namely, increased *Ucp1* and *Cidea* gene expression—in the group of CR-MI animals, albeit solely in the males. This agrees with the browning-stimulating action of MI supplementation in a strong connection with BDNF signaling. In line with this, we previously described that the CR male animals treated with MI during lactation displayed ameliorated insulin responsiveness capacity in the rWAT [13]. 

Changes in the gene expression pattern in the BAT associated with MI treatment were also sex-dependent but less marked than in the other tissues studied. It is noteworthy that the CR females treated with MI during suckling showed a greater BAT weight in adulthood compared to those treated with the vehicle, accompanied by the normalization of specific UCP1 protein levels, which were significantly diminished in the CR females treated with the vehicle. In this sense, it is worth mentioning that the CR females that received MI during lactation exhibited higher *Lepr* mRNA levels at 25 days, which suggests that they are more sensitive to leptin and, therefore, to the BAT response to this hormone. These results agree with the proposed leptin-sensitizing effect of MI ingested during suckling in conjunction with the activation of BDNF signaling at the gene expression level in the BAT.

In short, in the present work, we suggest, for the first time, that supplementing MI at physiological doses during suckling enhances BDNF signaling in offspring born to dams who experienced moderate calorie restriction during pregnancy, with a more pronounced effect observed in males. Increased circulating levels of BDNF were detected in the males at an early age, following MI treatment, and persisted into adulthood in the offspring of the calorie-restricted dams during gestation, both under a standard diet and after Western diet exposure. A potential association between the activation of BDNF and its signaling pathways and the enhanced insulin sensitivity and lipid profile in these animals has also been also proposed. This is evidenced by the promotion of anorexigenic pathways in the hypothalamus, along with an upregulation of browning markers and increased glucose uptake capacity in white and brown adipose tissue depots. The current findings pinpoint the BDNF pathway as one of the mechanisms contributing to the positive metabolic programming associated with MI supplementation during lactation in rats. More studies are required to further explore this mechanism.

## Figures and Tables

**Table 1:** Weight-related parameters at 25 days.

			MALE			FEMALE			3-Way
			V	MI	2-Way	V	MI	2-Way	
Body weight	g	CON	63.0 ± 1.4	64.7 ± 1.3	R	62.5 ± 1.2	59.8 ± 1.3	R	R
		CR	57.2 ± 2.3 #	57.1 ± 2.6 #		58.9 ± 1.9	54.9 ± 2.5		
Body fat	%	CON	9.77 ± 0.34	9.9 ± 0.36	-	9.85 ± 0.17	9.58 ± 0.21	-	R
		CR	9.20 ± 0.59	8.68 ± 0.62		9.40 ± 0.46	8.53 ± 0.51		
White adipose tissue	Inguinal (g)	CON	0.701 ± 0.034	0.787 ± 0.068	R	0.845 ± 0.068	0.775 ± 0.037	R	R
		CR	0.648 ± 0.060	0.620 ± 0.053		0.679 ± 0.064	0.587 ± 0.064 #		
	Retroperitoneal (g)	CON	0.140 ± 0.013	0.135 ± 0.016	-	0.110 ± 0.012	0.114 ± 0.014	R	S, R
		CR	0.110 ± 0.010	0.109 ± 0.008		0.093 ± 0.009	0.079 ± 0.012		
Brown adipose tissue	g	CON	0.270 ± 0.024	0.288 ± 0.037	-	0.275 ± 0.032	0.258 ± 0.025	-	-
		CR	0.258 ± 0.023	0.250 ± 0.023		0.254 ± 0.022	0.252 ± 0.018		

Weight-related parameters of the offspring of controls (CON) and calorie-restricted dams during gestation (CR) treated with vehicle (V) or myo-inositol (MI) during the suckling period, at the age of 25 days. Data are means ± SEM. Statistics: Three-way ANOVA was conducted to investigate the impacts of gestational calorie restriction (R), lactational myo-inositol treatment (M), and/or sex (S). Within each sex, two-way ANOVA was used to assess the effects of R and/or M. Student’s *t*-test was used for individual group comparisons: #, different compared to their corresponding control group.

**Table 2:** Weight-related parameters at 7 months.

			MALE			FEMALE			3-Way
			V	MI	2-Way	V	MI	2-Way	
Body weight	g	CON	567 ± 15	595 ± 10	-	303 ± 6	301 ± 9	-	S
		CR	559 ± 17	557 ± 15		293 ± 8	314 ± 11		
Body fat	%	CON	25.1 ± 0.8	22.5 ± 1.5	-	22.3 ± 1.2	21.9 ± 1.8	-	-
		CR	24.1 ± 2.0	24.0 ± 1.4		23.2 ± 1.8	24.7 ± 1.9		
White adipose tissue	Inguinal (g)	CON	21.7 ± 1.6	21.7 ± 2.1	-	7.79 ± 0.46	7.27 ± 0.81	-	S
		CR	21.6 ± 2.0	21.5 ± 2.2		7.59 ± 0.47	8.63 ± 0.77		
	Retroperitoneal (g)	CON	22.5 ± 1.5	20.9 ± 2.7	-	8.28 ± 0.49	7.08 ± 0.93	-	S × R
		CR	21.2 ± 2.1	19.7 ± 1.0		8.87 ± 0.93	8.76 ± 0.92		
Brown adipose tissue	g	CON	1.06 ± 0.06	1.19 ± 0.15	R	0.689 ± 0.026	0.659 ± 0.072	R	S × R
		CR	0.816 ± 0.050 #	0.885 ± 0.052		0.709 ± 0.034	0.836 ± 0.008 *		

Weight-related parameters of the offspring of controls (CON) and calorie-restricted dams during gestation (CR) treated with vehicle (V) or myo-inositol (MI) during the suckling period, at the age of 7 months. Data are means ± SEM. Statistics: Three-way ANOVA was conducted to investigate the impacts of gestational calorie restriction (R), myo-inositol treatment (M), and/or sex (S). Within each sex, two-way ANOVA was used to assess the effects of R and/or M. Student’s *t*-test was used for individual group comparisons: *, different from their corresponding vehicle-supplemented group; #, different compared to their corresponding control group.

## Data Availability

The data supporting the present outcomes are available upon reasonable request from the corresponding author due to privacy.

## Supplementary Materials

The following supporting information can be downloaded at: https://www.mdpi.com/article/10.3390/nu16070980/s1, Figure S1: Expression levels of *Insr* and *Lepr* in the hypothalamus at 7 months; Figure S2: Expression levels of *Ucp1* in brown adipose tissue at 25 days and 7 months. Figure S3: Bands of proteins analyzed by Western Blot.
