# Supplementary material for: Brain-Derived Neurotrophic Factor as a Potential Mediator of the Beneficial Effects of Myo-Inositol Supplementation during Suckling in the Offspring of Gestational-Calorie-Restricted Rats
Source: Nutrients. 2024 Mar 27;16(7):980. doi: 10.3390/nu16070980 (PMC11013066; doi:10.3390/nu16070980)
Supplement: Supplementary file 1 [file nutrients-16-00980-s001.zip › nutrients-2916670-supplementary.pdf]

Figure S1. Expression levels of *Insr* and *Lepr* in hypothalamus at 7 months.

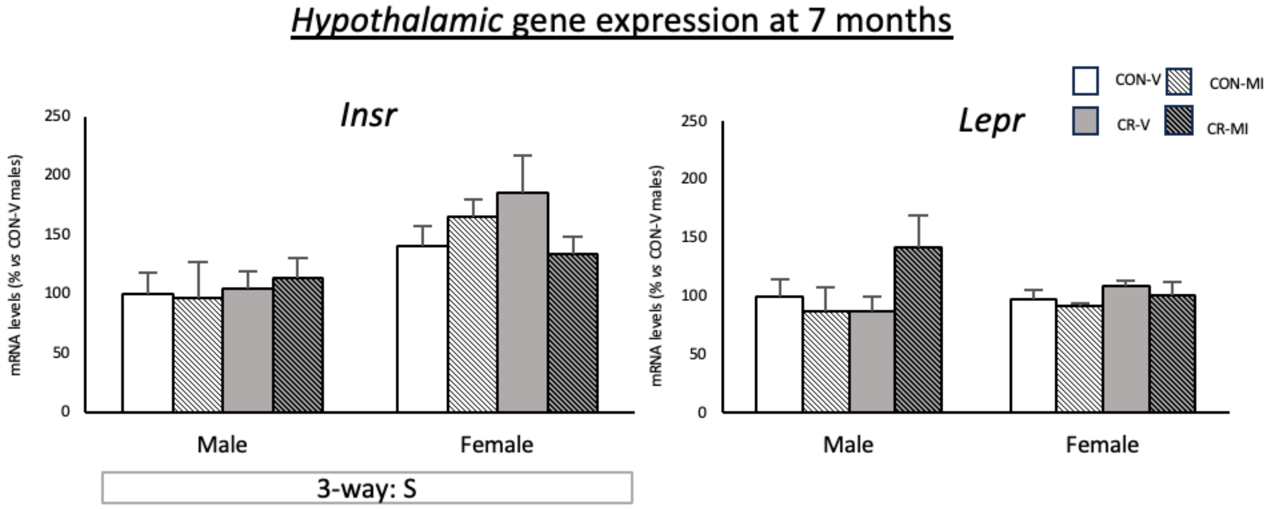

Expression level of *Insr* and *Lepr* in hypothalamus of the offspring of controls (CON) and calorie-restricted dams during gestation (CR) treated with vehicle (V) or myo-inositol (MI) during the suckling period, at the age of 7 months. mRNA levels are expressed as a percentage of the value of CON-V male animals. Data are mean ± SEM. Statistics: Three-way ANOVA was performed to analyze the effects of sex (S), gestational calorie restriction (R) and/or myo-inositol treatment during lactation (M). In each sex, two-way ANOVA was performed to analyze the effects of calorie restriction and/or myo-inositol supplementation. Single comparisons between groups were performed by Student's t-test: *, different from their respective vehicle supplemented group; #, different from their respective control group.

Figure S2. Gene expression levels of *Ucp1* in brown adipose tissue at 25 days-old and 7 months.

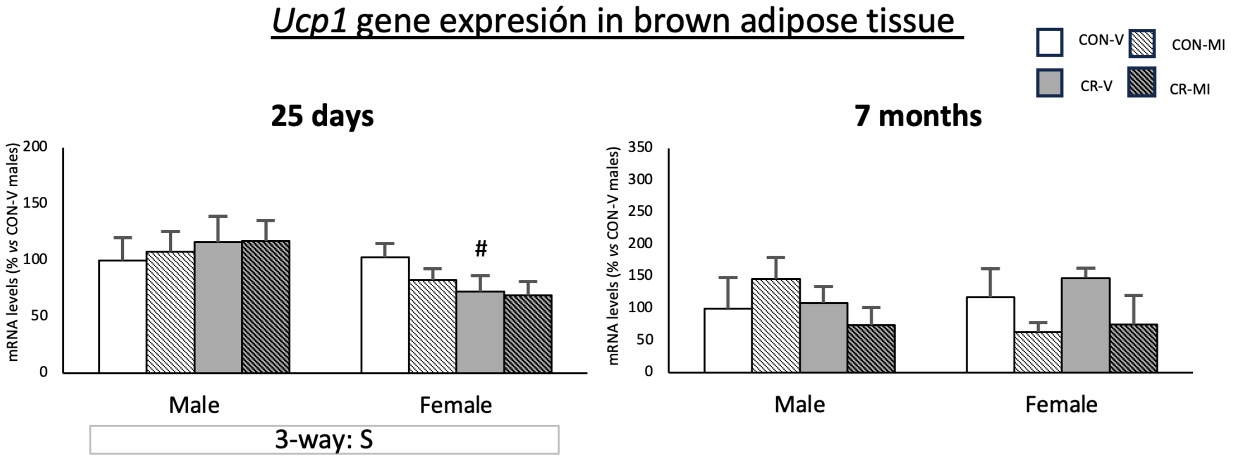

Expression level of *Ucp1* in brown adipose tissue of the offspring of controls (CON) and calorie-restricted dams during gestation (CR) treated with vehicle (V) or myo-inositol (MI) during the suckling period, at the age of 25 days and 7 months. mRNA levels are expressed as a percentage of the value of CON-V male animals. Data are mean ± SEM. Statistics: Three-way ANOVA was performed to analyze the effects of sex (S), gestational calorie restriction (R) and/or myo-inositol treatment during lactation (M). In each sex, two-way ANOVA was performed to analyze the effects of calorie restriction and/or myo-inositol supplementation. Single comparisons between groups were performed by Student's t-test: *, different from their respective vehicle supplemented group; #, different from their respective control group.

Figure S3. Bands of each protein of interest analysed by Western Blot

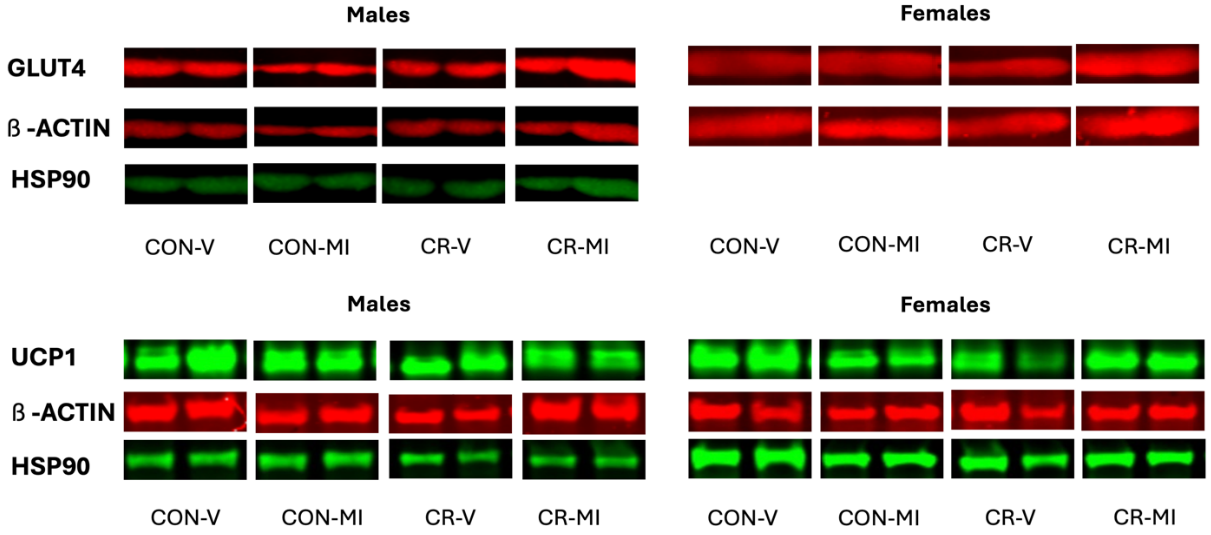

Representative bands of each protein of interest (GLUT4 and UCP1) and reference proteins (ß-Actin and/or HSP90) for each animal group are shown. They have been cut from the same membrane and grouped together in the image. Abbreviations: control (CON), gestational calorie-restricted (CR), vehicle (V), myo-inositol (MI).
